# Retrospective Correlation between First Drug Treatment Duration and Survival Outcomes in Sequential Treatment with Regorafenib and Trifluridine/Tipiracil in Refractory Metastatic Colorectal Cancer: A Real-World Subgroup Analysis

**DOI:** 10.3390/cancers15245758

**Published:** 2023-12-08

**Authors:** Carlo Signorelli, Mario Giovanni Chilelli, Diana Giannarelli, Michele Basso, Maria Alessandra Calegari, Annunziato Anghelone, Jessica Lucchetti, Alessandro Minelli, Lorenzo Angotti, Ina Valeria Zurlo, Marta Schirripa, Cristina Morelli, Emanuela Dell’Aquila, Antonella Cosimati, Donatello Gemma, Marta Ribelli, Alessandra Emiliani, Domenico Cristiano Corsi, Giulia Arrivi, Federica Mazzuca, Federica Zoratto, Maria Grazia Morandi, Fiorenza Santamaria, Rosa Saltarelli, Enzo Maria Ruggeri

**Affiliations:** 1 Medical Oncology Unit, Belcolle Hospital, ASL Viterbo, 01100 Viterbo, Italy; 2 Biostatistics Unit, Scientific Directorate, Fondazione Policlinico Universitario Agostino Gemelli, IRCCS, 00168 Rome, Italy; 3 Unit of Medical Oncology, Comprehensive Cancer Center, Fondazione Policlinico Universitario Agostino Gemelli, IRCCS, 00168 Rome, Italy; 4 Division of Medical Oncology, Policlinico Universitario Campus Bio-Medico, 00128 Rome, Italy; 5 Medical Oncology, “Vito Fazzi” Hospital, 73100 Lecce, Italy; 6 Medical Oncology Unit, Department of Systems Medicine, Tor Vergata University Hospital, 00133 Rome, Italy; 7 Medical Oncology 1, IRCCS Regina Elena National Cancer Institute, 00144 Rome, Italy; 8 Medical Oncology Unit, ASL Frosinone, 03039 Sora (FR), Italy; 9 Medical Oncology Unit, Isola Tiberina Hospital-Gemelli Isola, 00186 Rome, Italy; 10 Department of Clinical and Molecular Medicine, Oncology Unit, Sant’ Andrea University Hospital, Sapienza University of Rome, 00189 Rome, Italy; 11 Medical Oncology Unit, ASL Latina, 04100 Latina, Italy; 12 Medical Oncology Unit, San Camillo de Lellis Hospital, ASL Rieti, 02100 Rieti, Italy; 13 UOC Oncology A, Policlinico Umberto I, 00185 Rome, Italy; 14 Experimental Medicine, Network Oncology and Precision Medicine, Department of Experimental Medicine, Sapienza University of Rome, 00189 Rome, Italy; 15 UOC Oncology, San Giovanni Evangelista Hospital, ASL RM5, 00019 Tivoli (RM), Italy

**Keywords:** metastatic colorectal cancer, regorafenib, trifluridine/tipiracil, first drug treatment duration, sequential treatment, third-line therapy, real-world study

## Abstract

**Simple Summary:**

Third-line or further treatments are rarely given to patients with refractory mCRC. In this regard, two noteworthy innovative therapy options, with varying toxicity profiles and statistically significant improvements in overall survival (OS), progression-free survival (PFS), and disease control, are regorafenib (R) and trifluridine/tipiracil (T). This study is a subset analysis of a larger retrospective study that we have already published, with the aim of evaluating patient outcomes when R and T were administered in that order. The purpose of this analysis was to evaluate the relationship between survival outcomes and the first drug treatment duration (<3 months, 3 to <6 months, and ≥6 months) in patients who had received the regorafenib-to-trifluridine/tipiracil sequence or vice versa. Our substudy found that administering R three to six months prior to T can prolong both OS and PFS in comparison to the opposite sequence.

**Abstract:**

*Background*: Patients with refractory metastatic colorectal cancer (mCRC) rarely receive third-line or further treatment. In this context, regorafenib (R) and trifluridine/tipiracil (T) are two important novel therapeutic choices with statistically significant increases in overall survival (OS), progression-free survival (PFS), and disease control, with different toxicity profiles. This study is a subgroup analysis of our larger retrospective study, already published, whose objective was to assess the outcomes of patients when R and T were given sequentially. *Patients and Methods*: The study involved thirteen Italian cancer centers on a 10-year retrospective observation (2012–2022). In this subgroup analysis, we focused our attention on the correlation between the first drug treatment duration (<3 months, 3 to <6 months and ≥6 months) and survival outcomes in patients who had received the sequence regorafenib-to-trifluridine/tipiracil, or vice versa. *Results*: The initial study included 866 patients with mCRC who received sequential T/R, or R/T, or T or R alone. This analysis is focused on evaluating the impact of the duration of the first treatment in the sequence on clinical outcomes (OS, PFS) and includes 146 and 116 patients of the T/R and R/T sequences, respectively. Based on the duration of the first drug treatment, subgroups for the T/R sequence included 27 patients (18.4%) who received T for <3 months, 86 (58.9%) treated for 3 to <6 months, and 33 (22.6%) treated for ≥6 months; in the reverse sequence (R as the first drug), subgroups included 18 patients (15.5%) who received their first treatment for <3 months, 62 (53.4%) treated for 3 to <6 months, and 35 (31.0%) treated for ≥6 months. In patients who received their first drug treatment for a period of 3 to <6 months, the R/T sequence had a significantly longer median OS (13.7 vs. 10.8 months, *p* = 0.0069) and a longer median PFS (10.8 vs. 8.5 months, *p* = 0.0003) than the T/R group. There were no statistically significant differences between groups with first drug treatment durations of <3 months and ≥6 months. *Conclusions*: Our analysis seems to suggest that the administration of R for a period of 3 to <6 months before that of T can prolong both OS and PFS, as compared to the opposite sequence.

## 1. Introduction

Colorectal cancer (CRC) is the second most common cancer to cause death in the United States and the fourth most common cancer diagnosed globally. Colon and rectal cancer cases are expected to increase by 106,970 and 46,050, respectively, in 2023. The combined death toll from colon and rectal cancers is anticipated to be 52,550 in the same year [1,2]. In 33% of instances of CRC, metastases will manifest themselves, either at presentation or during follow-up, according to reports on 5-year overall survival (OS) for metastatic CRC (mCRC) [3]. Despite these alarming data, CRC mortality has been decreasing for years, and it is now more than 50% lower than it was at its highest. These reductions in CRC incidence and mortality are assumed to be the results of earlier cancer detection through screening, cancer prevention, and improved treatment options. 

The molecular properties of the tumor, the therapy target, the patient’s overall health, the tumor load, and the clinical course are some of the factors that affect the treatment options. Based on the patient’s features, the tumor’s sidedness, molecular tests like RAS and BRAF mutations, and the Eastern Cooperative Oncology Group Performance Status (ECOG PS), a treatment plan is chosen. Systemic therapy for mCRC often starts with chemotherapy regimens, combining a fluoropyrimidine with either oxaliplatin or irinotecan. These cytotoxic backbones frequently incorporate biologic drugs. In particular, chemotherapy can be combined with monoclonal antibodies that target either the vascular endothelial growth factor pathway, like bevacizumab, or the epidermal growth factor receptor, like cetuximab or panitumumab. The latter class of antibodies is only effective in patients with RAS and BRAF wild-type tumors. 

For mCRC that has advanced with the aforementioned therapy, two oral drugs have been researched and approved. Regorafenib (R) and trifluridine/tipiracil (T), regarded as later-line standard treatments, have been found to prolong survival for patients with resistant metastatic colorectal cancer; however, not all patients experience positive outcomes [4,5,6,7,8,9]. The phase III CORRECT trial, which compared R with placebo in patients with previously treated mCRC who had progressed beyond their last given standard therapy, provided the basis for the FDA’s 2012 approval of R, an oral multikinase inhibitor [5]. Overall survival (OS) was shown to be significantly longer in patients receiving R, in comparison to those receiving a placebo (mOS, 6.4 vs. 5.0 months; hazard ratio (HR), 0.77; *p* = 0.005) [5]. The oral chemotherapy drug T consists of tipiracil, an inhibitor of thymidine phosphorylase, and trifluridine, a cytotoxic agent. Based on the findings of the phase III RECOURSE trial, which compared T with placebo in patients with mCRC who had received at least two prior standard chemotherapy regimens, T was approved in 2015 for the same group of patients for whom R was approved [6]. When comparing patients treated with T to those given a placebo, this trial demonstrated a significant improvement in median OS (mOS, 7.1 vs. 5.3 months; HR, 0.68; *p* < 0.001) [6].

However, because each case is unique and there are numerous molecular subtypes, it can be challenging to evaluate treatment options. In addition, given the wide range of tolerance profiles and the modest improvements in OS and PFS, T and R are still sometimes thought of as being of little clinical significance [10,11,12,13,14,15,16,17,18,19,20,21,22,23,24,25,26,27,28,29,30]. 

Our recent investigation demonstrates the efficiency and controllable side effects of late-line administration of T and R for the treatment of metastatic colorectal cancer in a clinical scenario. According to our research, giving R before T can aid in extending both PFS and OS, even without tumor reduction [31]. Although it has not yet been determined which agent should be given first, we want to offer additional analyses of sequential R and T treatments, including results based on the treatment duration of the first drug administered.

## 2. Patients and Methods

The current analysis is based on data from our prior study, which was recently published. Thirteen Italian cancer centers participated in our 10-year (2012–2022) retrospective observational study. The specifics of the study’s design have been reported. In summary, T or R alone, sequential T/R, or sequential R/T were administered to 866 patients with mCRC in the original research. In contrast to the opposite sequence, we found that the R/T sequence resulted in significantly longer OS (15.9 vs. 13.9 months, *p* = 0.0194) and PFS (11.2 vs. 8.8 months, *p* = 0.0005). However, non-sequential R or T administration had similar effects on survival [31]. 

### 2.1. Study Design

**Figure 1:**
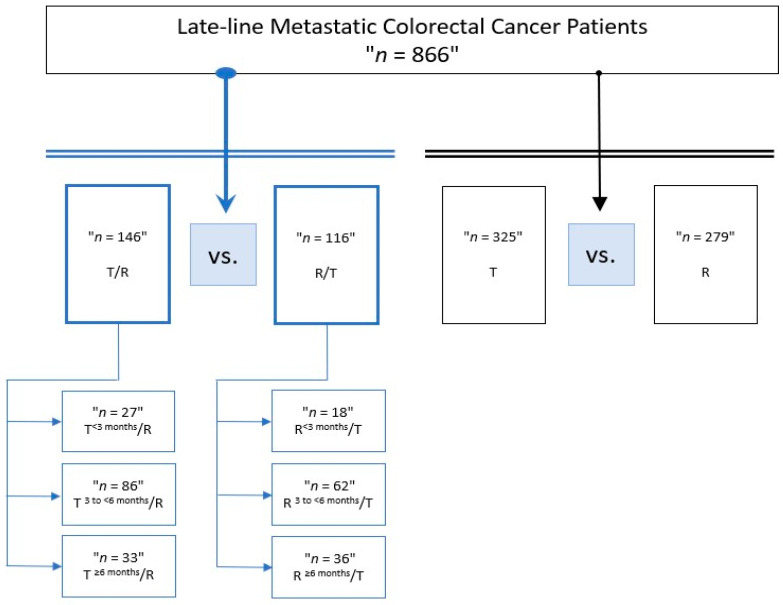
Study design, with the division of groups according to the treatment duration of the first drug in the sequential treatment. Abbreviations: T, trifluridine/tipiracil; R, regorafenib.

In the present retrospective substudy, we focused our attention on the correlation between the first drug treatment duration (3 months, 3 to <6 months and ≥6 months) and survival outcomes in patients who received sequential treatment with T followed by R, or vice versa, for 3rd- and 4th-line therapy in real world clinical practice. Eligible patients with metastatic colorectal cancer (mCRC) were those who were at least eighteen years old and had progressed following at least two previous regimens of standard chemotherapy with fluoropyrimidine, irinotecan, oxaliplatin, or anti-VEGF (bevacizumab and aflibercept) or anti-EGFR (cetuximab or panitumumab) antibodies. Patients were followed up until the date of death or loss to follow up. Figure 1 displays the study’s design.

This study’s main goal was to observe the routine clinical practice use of sequential treatment with regorafenib and trifluridine/tipiracil and vice versa, assessing its effectiveness according to the treatment duration of the first drug administered in the sequence. The primary endpoints were overall survival (OS), which was defined as the time from the beginning of the first treatment (R or T) to death from any cause during the second treatment (T or R) and progression-free survival (PFS), which was defined as the time from the start of the first treatment (R or T) to the progression of the disease or death from any cause during the second treatment (T or R). At the time of the last follow-up, patients who were not experiencing an event were censored. Our secondary goal was to find potential predictive response factors for survival.

The study was completed in accordance with the Helsinki Declaration. All data were anonymized to protect sensitive information, and patients were only identifiable by their initials and a number. The lead investigator was the data manager and had access to the whole database, as required by law. To exclude any potential selection bias, the current analysis included all patients undergoing consecutive R/T and T/R. Endpoints for the investigations were established in order to lessen the chance of distortion bias. Because the study was retrospective, we should be mindful that the results presented should be taken as exploratory. Patient data confidentiality was upheld despite informed authorization being waived due to the nature of the retrospective study. 

### 2.2. Statistical Analysis

Descriptive statistics were used to compile the relevant data. Using the Chi-square and Fisher exact tests, potential associations were evaluated. PFS and OS were calculated using the Kaplan–Meier product limit method, and differences among subgroups were assessed using the log-rank test. *p* ≤ 0.05 was used to determine significance. SPSS Statistics software, version 21.0, was used to conduct all of the statistical analyses.

## 3. Results

### 3.1. Baseline Characteristics

Of 866 patients enrolled in the former study [31], 146 were prescribed T first, followed by R (T/R), and 116 received R first, followed by T (R/T); these were investigated for the current analysis. No other therapies were administered between the investigated treatments. The overall median follow-up time was 10.8 months (95% confidence interval (CI) = 9.4–51.3) for the T/R sequence and 13.7 months (95% CI = 12.5–73.0) for the R/T group. In the T/R sequence, T and R had median treatment times of 4.1 (range 1.9–29.0) and 3.4 (range 0.5–28.1) months, respectively. In the reverse sequence, T had a median duration of 3.7 (range 0.3–22.6) months, as opposed to 4.4 (range 0.6–45.2) months for R. 

Subgroups based on the duration of T first included 27 patients who received T for <3 months, 86 treated for 3 to <6 months, and 33 treated for ≥6 months for the T/R sequence; in the reverse sequence, based on the duration of R first, we observed 18 patients who received R for <3 months, 62 treated for 3 to <6 months and 36 treated for ≥6 months. 

There were some differences in baseline demographics and clinical characteristics across all subgroups, including the prevalence of patients who were <70 years old, a primary tumor on the left side, ECOG PS 0–1, liver + other metastases, the administration of doublet chemotherapy, male sex, and anti-VEGF use, regardless of T or R treatment duration. The cohort with a first drug treatment duration of 3 to <6 months presented the highest number of patients (Table 1). 

### 3.2. Efficacy Outcomes 

**Figure 2:**
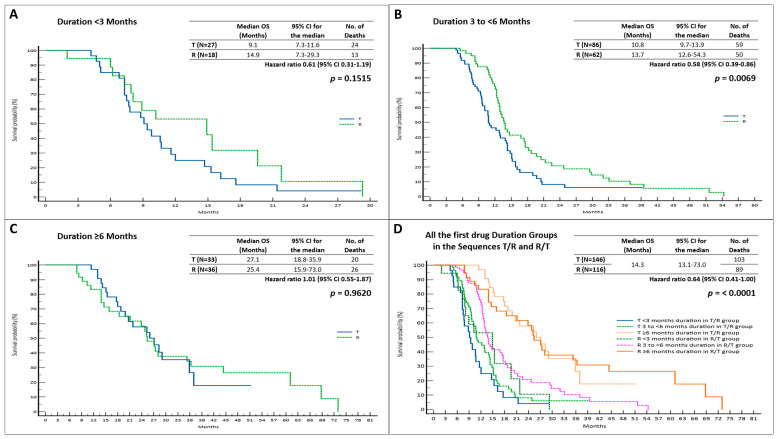
Overall survival based on first drug treatment duration in R/T and T/R sequences; (**A**). OS when first drug treatment duration was <3 months; (**B**). OS when first drug treatment duration was 3 to <6 months; (**C**). OS when first drug treatment duration was ≥6 months; (**D**). OS among all groups. Abbreviations: OS, overall survival; 95% CI, 95% confidence interval; T, trifluridine/tipiracil; R, regorafenib.

In patients who were given T first in the T/R sequence, we observed a longer, but not statistically significant, median OS (mOS) when the duration of T was ≥6 months (*p* = 0.9620). There were no benefits observed in the other two groups (Figure 2, Table 2). 

**Figure 3:**
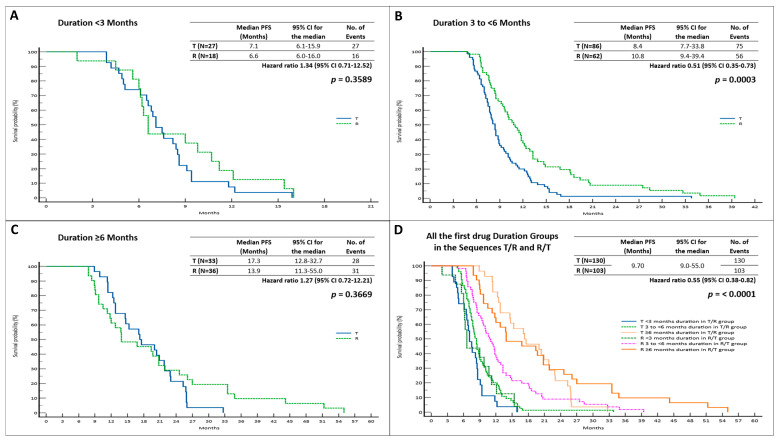
Progression-free survival based on first drug treatment duration in R/T and T/R sequences; (**A**). PFS when first drug treatment duration was <3 months; (**B**). PFS when first drug treatment duration was 3 to <6 months; (**C**). PFS when first drug treatment duration was ≥6 months; (**D**). PFS among all groups. Abbreviations: PFS, progression-free survival; 95% CI, 95% confidence interval; T, trifluridine/tipiracil; R, regorafenib.

In terms of mPFS, we observed a statistically insignificant advantage when T was administered for <3 months (*p* = 0.3589) or ≥6 months (*p* = 0.3969) (Figure 3, Table 2). 

In patients who received R for 3 to <6 months, followed by T, we observed a significantly longer mOS (13.7 months; 95% CI = 12.6–54.3; HR = 0.58 vs. 10.8 months; 95% CI = 9.7–13.9; HR = 1.72, *p* = 0.0069) compared to that observed in the reverse sequence (Figure 2). In addition, we documented insignificant mOS advantages for the other two subgroups (Figure 2, Table 2). 

In the R/T group, mPFS was considerably longer than that observed for the T/R sequence (10.8 months; 95% CI = 9.4–39.4; HR = 0.51 vs. 8.4 months; 95% CI = 7.7–33.8; HR = 1.96, *p* = 0.0003) for patients who received R first for 3 to <6 months. The results for the remaining two subgroups, in terms of mPFS, were not statistically significant (Figure 3), (Table 2). 

In the first draft of the manuscript, we included the overall response rate and the disease control rate. Due to the lack of significance in the results, we opted to focus solely on survival outcomes.

### 3.3. Prognostic Factors for OS and PFS

The division into the six groups is significant, even adjusted for age, ECOG PS, and metastatic sites. These variables were chosen for multivariate analysis for OS and PFS in all groups and, specifically, in the R 3 to <6 months/T and in the T 3 to <6 months/R cohorts. These results are shown in Table 3 and Table 4, respectively. In the multivariate analysis, we observed a statistically significant correlation between ECOG PS and OS or PFS (*p* = 0.014 and *p* = 0.013, respectively). The same correlation was found in the multivariate analysis of the two subgroups with a first drug treatment duration of 3 to <6 months (*p* = 0.027 and *p* = 0.033, respectively). 

Univariate survival Cox regression analysis was performed in the R 3 to <6 months/T group. In this cohort, we observed statistically significant relationships between ECOG PS and OS (HR = 0.44; 95% CI = 0.20–0.98; *p* = 0.0439) and PFS (HR = 0.67; 95% CI = 0.38–1.17; *p* = 0.0251) (Table 5). 

## 4. Discussion

It is generally accepted that there are no differences in the conventional parameters of efficacy, whichever of the two agents (regorafenib or trifluridine/tipiracil) is used first, while some reports suggested that patients who received T after R showed a trend towards a longer PFS than those who received T before R [31,32]. The sequencing of R before T, or vice versa, does not yet have sufficient clinical evidence to support it in real-world practice. Due to the similar indications and administration methods between R and T, this knowledge could be relevant. 

In line with our previous research, we tried to investigate T and R treatment sequencing further, for metastatic colorectal cancer that had relapsed or was refractory [16,19]. We hypothesized that therapy with R prior to that with T could result in improved survival results compared to reverse sequencing, based on interesting data from our previous real-world experience [31].

The effects of the first drug treatment duration on survival among patients with mCRC who received T and R sequentially, and vice versa, were the main focus of this subgroup analysis. This is the first study on this topic in the literature, as far as we are aware. Furthermore, we examined survival outcomes within the patient subgroups that had the best OS and PFS findings. 

In our real-world study, we found that the R 3 to <6 months/T cohort had a longer mOS compared to the T 3 to <6 months/R group (13.7 months vs. 10.8 months, respectively; *p* = 0.0069). In the same group, there was a statistically significant advantage in mPFS (10.8 months vs. 8.4 months; *p* = 0.0003). Regarding the other groupings, no noteworthy results were found. The fact that the R 3 to <6 months/T sequence contained the only statistically meaningful survival data we could find may still come as a surprise. Though this is only conjecture, it is possible that this is because this group includes more than half of the patients in the two 3 to <6 months sequences. The results we came to in our earlier articles [16,19,31] are further supported by this finding. However, the lack of benefit seen in the <3 months and ≥6 months groups may be explained by the limited sample sizes of patients.

On both multivariate and univariate analyses, ECOG PS was the only variable that correlated statistically with survival outcomes. Additionally, univariate analysis revealed that patients in the R 3 to <6 months/T group with an ECOG PS = 0 had a significantly higher survival benefit than patients with an ECOG PS = 1, and particularly when compared to patients with an ECOG PS = 2. There was no significant numerical difference in the performance statuses of the two other subgroups with first treatment durations ranging from 3 to less than 6 months and ≥6 months. Presently, no studies have been conducted to compare these results with survival outcomes based on the duration of the first drug treatment duration in this type of sequencing.

Patients with only liver metastases who were given the R/T sequence with a first drug treatment duration of 3 to <6 months had a 3- to 4-month longer mOS than patients with liver + other metastases. However, in the univariate analysis of the R 3 to <6 months/T group, the metastatic locations did not reach statistical significance, but a positive trend was noted. Regardless of the first drug’s treatment duration, on the basis of the multivariate analysis, we could state that we identified a single survival predictor for all groups receiving sequential treatment (T/R or R/T), with the R 3 to <6 months/T cohort being the most impacted. As a result, we suggest that patients with an ECOG PS of 0 or 1 will benefit the most from R/T treatment if the R treatment duration is between 3 and <6 months.

The study eligibility criteria between our observational study and other published studies varied. Patients had to have received standard therapies in the CORRECT study, and 49% of those receiving regorafenib had received at least four different types of previous therapies [5]. Patients had to have undergone at least two standard chemotherapy regimens prior to enrolling in the RECOURSE trial; 60% of FTD/TPI-treated participants had undergone at least four lines of treatment prior to enrollment [6]. Since our analysis was a real-world study that more closely mirrored clinical practice and experience, there were no particular limitations regarding prior therapies. 

Determining the optimal sequential treatment for a patient with metastatic colorectal cancer in the third line of therapy and beyond is a changing field. The choice is undoubtedly influenced by the toxicity profiles of the two drugs, the patient’s performance status, the toxicity of prior therapies, and the nature and extent of the disease, but the research data should also not be disregarded. 

For instance, adding bevacizumab to T has been recently shown to improve OS and PFS when compared to a placebo in the phase 3 randomized SUNLIGHT trial [27,28]. 

As we mentioned in our previous paper [31], it may be more effective to administer R before T. According to the findings of this subgroup study, the effectiveness of R appears to be significantly higher when provided for between three and six months. As it is impossible to predict, in advance, how long the first drug in the treatment sequence will be administered, further research is needed to identify prognostic and/or predictive factors of treatment response.

T/R or R/T are very interesting in daily clinical practice when the first and second lines of therapy fail, if we consider the switching of the two drugs’ distinct mechanisms of action. We can provide the patient options for sequencing, but every time we do so, we question where R or T should go. It is more comparable to a salvage line if there are not many options in this case. Moreover, our observations indicate that the overall survival of these patients is improving. It is likely that this is just the outcome of selection. 

It is important to recognize the limitations of the analysis presented here. Since this is a retrospective study of real-world practice, the most significant limitation, in our opinion, is that the statistically significant OS and PFS benefits observed only in the R 3 to <6 months/T cohort could be a result of numerous uncontrollable factors (e.g., small numbers of subgroups, clinical disease progression, the oncologist’s potential to delay the duration of therapy given the limited number of additional treatment options, etc.). This is not to say that the information is uninteresting. 

In our retrospective study, we attempted to be helpful to real-world practice in terms of drug sequencing and further, especially in terms of the potential impact of the duration of the first agent in the sequence on survival. In any case, we believe that the most essential thing is to have the option of providing care for patients, even in the later lines of therapy. And in this setting, R and T have a role and a purpose. 

## 5. Conclusions

In conclusion, our analysis confirms the effectiveness of late-line administration of T and R for the treatment of mCRC in a real-world setting, but we still need to learn more about them, such as their treatment durations in sequential use.

In our substudy, we found that, compared to the opposite sequence, the administration of R for a duration of 3 to 6 months before the administration of T can contribute to prolonging both OS and PFS. 

Our insights, however, attest to the necessity for additional investigation in this area to support a patient’s ability to live with a sufficient quality of life by focusing more on preventing the progression of the disease, rather than on its observable responses.

## Figures and Tables

**Table 1:** Patients characteristics at baseline. Abbreviations: T, trifluridine/tipiracil; R, regorafenib; pts, patients; MSI, microsatellite instability; ECOG PS, Eastern Cooperative Oncology Group Performance Status; CT, chemotherapy.

			Duration of First Drug Treatment					
	Overall		<3 Months		3 to <6 Months		≥6 Months	
	T/R	R/T	T/R	R/T	T/R	R/T	T/R	R/T
N (%)	146 (100)	116 (100)	27 (100)	18 (100)	86 (100)	62 (100)	33 (100)	36 (100)
Median age, years (range)	69 (30–84)	66 (43–84)	64 (48–80)	67 (51–82)	70 (30–83)	68 (43–83)	69 (49–84)	68 (53–82)
pts ≥ 70 years								
yes	67 (45.9)	42 (36.3)	9 (33.3)	7 (38.8)	44 (51.2)	20 (32.3)	14 (42.4)	15 (41.7)
no	79 (54.1)	74 (63.7)	18 (66.7)	11 (61.2)	42 (48.8)	42 (67.7)	19 (57.6)	21 (58.3)
Gender								
Female	51 (35.0)	44 (38.0)	10 (37.0)	4 (22.3)	29 (33.7)	15 (24.2)	12 (36.4)	25 (69.4)
Male	95 (65.0)	72 (62.0)	17 (63.0)	14 (77.7)	57 (66.3)	47 (75.8)	21 (63.6)	11 (30.6)
RAS status								
Wild type	49 (33.5)	42 (36.2)	6 (22.3)	6 (33.4)	31 (36.0)	21 (33.9)	13 (39.4)	15 (41.7)
Mutant type	90 (61.7)	67 (57.7)	21 (77.7)	10 (55.5)	49 (57.0)	37 (59.6)	20 (60.6)	20 (55.6)
Unknown	7 (4.8)	7 (6.1)	0 (0.0)	2 (11.1)	6 (7.0)	4 (6.5)	0 (0.0)	1 (2.7)
Primary tumor location								
Right side	52 (35.6)	34 (29.3)	8 (29.6)	4 (22.3)	37 (43.0)	18 (29.0)	7 (21.2)	12 (33.3)
Left side	94 (64.4)	82 (70.7)	19 (70.4)	14 (77.7)	49 (57.0)	44 (71.0)	26 (78.8)	24 (66.7)
MSI								
Yes	3 (2.0)	4 (3.4)	0 (0.0)	2 (11.1)	2 (2.3)	1 (1.6)	1 (3.0)	1 (2.7)
No	89 (61.0)	64 (55.2)	17 (63.0)	10 (55.5)	49 (57.0)	36 (58.0)	23 (69.7)	18 (50.0)
Unknown	54 (37.0)	48 (41.4)	10 (37.0)	6 (33.4)	35 (40.7)	25 (40.4)	9 (27.3)	17 (47.3)
PS ECOG								
0–1	128 (87.7)	108 (93.2)	24 (88.9)	16 (88.9)	75 (87.2)	57 (92.0)	29 (87.9)	35 (97.3)
2	18 (12.3)	8 (6.8)	3 (11.1)	2 (11.1)	11 (12.8)	5 (8.0)	4 (12.1)	1 (2.7)
Prior adjuvant therapy								
yes	46 (31.5)	43 (37.1)	7 (26.0)	4 (22.3)	22 (25.6)	22 (35.5)	17 (51.5)	17 (47.3)
no	100 (68.5)	66 (56.9)	20 (74.0)	14 (77.7)	64 (74.4)	40 (64.5)	16 (48.5)	19 (52.7)
Metastatic disease sites								
Liver only	23 (15.7)	14 (12.0)	4 (14.8)	1 (5.6)	16 (18.6)	7 (11.3)	3 (9.1)	6 (16.7)
Liver + other	80 (54.8)	51 (44.0)	17 (62.9)	9 (50.0)	46 (53.5)	29 (46.8)	17 (51.5)	13 (36.1)
Others	43 (29.5)	51 (44.0)	6 (22.3)	8 (44.4)	24 (27.9)	26 (41.9)	13 (39.4)	17 (47.2)
CT 1°line regimen								
Doublet chemotherapy	112 (76.8)	94 (81.1)	21 (77.7)	15 (83.3)	69 (80.2)	48 (77.4)	29 (87.8)	31 (86.1)
Triplet chemotherapy	16 (10.9)	9 (7.7)	6 (22.3)	1 (5.6)	9 (10.5)	7 (11.3)	2 (6.1)	1 (2.7)
Fluoropyrimdine alone	18 (12.3)	13 (11.2)	0 (0.0)	2 (11.1)	8 (9.3)	7 (11.3)	2 (6.1)	4 (11.2)
CT 2°line regimen								
Doublet chemotherapy	119 (81.6)	94 (81.1)	24 (88.9)	15 (83.3)	72 (83.7)	50 (80.6)	23 (69.7)	29 (80.6)
Triplet chemotherapy	2 (1.3)	2 (1.7)	1 (3.7)	1 (5.6)	1 (1.2)	0 (0.0)	0 (0.0)	1 (2.7)
Monochemotherapy	25 (17.1)	20 (17.2)	2 (7.4)	2 (11.1)	13 (15.1)	12 (19.4)	10 (30.3)	6 (16.7)
Biological agents 1°line								
Anti-EGFR use	43 (29.5)	30 (25.8)	4 (14.8)	3 (16.7)	29 (33.7)	15 (24.2)	10 (30.3)	12 (33.3)
Anti-VEGF use	72 (49.4)	65 (56.1)	18 (66.6)	10 (55.5)	38 (44.2)	34 (54.8)	17 (51.5)	19 (52.7)
None	31 (21.1)	21 (18.1)	5 (18.6)	5 (27.8)	19 (22.1)	13 (21.0)	6 (18.2)	5 (14.0)
Biological agents 2°line								
Anti-EGFR use	9 (6.1)	7 (6.0)	0 (0.0)	1 (5.6)	6 (7.0)	2 (3.2)	3 (9.1)	4 (11.2)
Anti-VEGF use	97 (66.5)	81 (69.9)	19 (70.4)	14 (77.7)	57 (66.3)	45 (72.6)	21 (63.6)	23 (63.8)
None	40 (27.3)	28 (24.1)	8 (29.6)	3 (16.7)	23 (26.7)	15 (24.2)	9 (27.3)	9 (25.0)

**Table 2:** Efficacy outcomes. Abbreviations: mos, months; OS, overall survival; mOS, median overall survival; PFS, progression-free survival; mPFS, median progression-free survival; 95% CI, 95% confidence interval; HR, hazard ratio; T, trifluridine/tipiracil; R, regorafenib.

	OS				PFS			
	mOS (mos)	95% CI	HR	*p*-Value	mPFS (mos)	95% CI	HR	*p*-Value
T ^<3 months^/R	9.1	7.3–11.6	1.10 0.92–1.32	0.1515	7.1	6.1–15.9	1.34 0.71–2.52	0.3589
R ^<3 months^/T	14.9	7.3–29.3	0.61 0.31–1.19		6.6	6.0–16.0	0.74 0.39–1.39	
T ^3 to <6 months^/R	10.8	9.7–13.9	1.72 1.16–2.56	0.0069	8.4	7.7–33.8	1.96 1.36–2.81	0.0003
R ^3 to <6 months^/T	13.7	12.6–54.3	0.58 0.39–0.86		10.8	9.4–39.4	0.51 0.35–0.73	
T ^≥6 months^/R	27.1	18.8–35.9	1.01 0.55–1.87	0.9620	17.3	12.8–32.7	1.27 0.72–2.21	0.3969
R ^≥6 months^/T	25.4	15.9–73.0	0.98 0.53–1.81		13.9	11.3–55.0	0.78 0.72–1.37	

**Table 3:** Multivariate analysis for OS and PFS. Abbreviations: T, trifluridine/tipiracil; R, regorafenib; OS, overall survival; PFS, progression-free survival; 95% CI, 95% confidence interval; ECOG PS, Eastern Cooperative Oncology Group Performance Status.

	OS		PFS	
	HR (95% CI)	*p*-Value	HR (95% CI)	*p*-Value
Treatment		<0.001		<0.001
R < 3 months/T	Reference		Reference	
R 3 to <6 months/T	0.68 (0.42–1.10)		0.57 (0.37–0.89)	
R ≥ 6 months/T	0.15 (0.08–0.29)		0.13 (0.07–0.23)	
T < 3 months/R	0.65 (0.32–1.29)		0.71 (0.37–1.36)	
T 3 to <6 months/R	0.40 (0.24–0.67)		0.29 (0.18–0.47)	
T ≥ 6 months/R	0.15 (0.08–0.28)		0.10 (0.06–0.19)	
Age		0.22		0.90
<70 years	Reference		Reference	
≥70 years	0.82 (0.61–1.12)		1.02 (0.77–1.34)	
ECOG PS		0.014		0.013
0	Reference		Reference	
1	0.76 (0.55–1.04)		1.22 (0.91–1.63)	
2	0.40 (0.21–0.75)		0.61 (0.37–1.01)	
Metastatic sites		0.071		0.21
Liver only	Reference		Reference	
Liver + other	1.39 (0.87–2.23)		0.97 (0.66–1.43)	
Others	0.98 (0.59–1.62)		0.75 (0.49–1.15)	

**Table 4:** Multivariate analysis for OS and PFS in the R ^3 to <6 months^/T and T ^3 to <6 months^/R groups. Abbreviations: T, trifluridine/tipiracil; R, regorafenib; OS, overall survival; PFS, progression-free survival; 95% CI, 95% confidence interval; ECOG PS, Eastern Cooperative Oncology Group Performance Status.

	OS		PFS	
	HR (95% CI)	*p*-Value	HR (95% CI)	*p*-Value
Treatment		0.013		0.001
T 3 to <6 months/R	Reference		Reference	
R 3 to <6 months/T	0.60 (0.40–0.89)		0.54 (0.37–0.78)	
Age		0.249		0.61
<70 years	Reference		Reference	
≥70 years	0.78 (0.52–1.18)		0.90 (0.62–1.31)	
ECOG PS		0.027		0.033
0	Reference		Reference	
1	0.82 (0.53–1.27)		1.26 (0.84–1.89)	
2	0.29 (0.11–0.71)		0.55 (0.27–1.10)	
Metastatic sites		0.373		0.59
Liver only	Reference		Reference	
Liver + other	1.47 (0.78–2.75)		0.99 (0.59–1.66)	
Others	1.17 (0.60–2.27)		0.82 (0.47–1.42)	

**Table 5:** Survival analysis in the R ^3 to <6 months^/T group, according to clinicopathological features. Abbreviations: OS, overall survival; mOS, median overall survival; PFS, progression-free survival; mPFS, median progression-free survival; 95% CI, 95% confidence interval; HR, hazard ratio; T, trifluridine/tipiracil; R, regorafenib; MSI, microsatellite instability.

Variables	OS				PFS			
	mOS (Months)	95% CI	HR (95% CI)	*p*-Value	mPFS (Months)	95% CI	HR (95% CI)	*p*-Value
Age				0.2877				0.4812
<70 years	14.3	12.8–54.3	0.70		11.3	8.8–39.4	0.80	
≥70 years	12.9	12.1–39.5	(0.37–1.33)		10.2	8.4–28.4	(0.45–1.45)	
Sex				0.8292				0.1731
Female	12.9	8.8–51.6	0.92		9.7	7.3–32.7	0.60	
Male	13.7	12.3–54.3	(0.47–1.80)		11.7	9.4–39.4	(0.29–1.24)	
RAS status				0.7623				0.9596
Wild type	17.6	10.8–39.5	0.90		11.5	7.3–32.7	0.98	
Mutant type	13.9	12.3–54.3	(0.48–1.69)		10.2	8.8–39.4	(0.54–1.78)	
Primary tumor location				0.6560				0.8442
Right side	13.1	8.3–17.6	0.85		10.1	6.8–34.9	0.94	
Left side	13.7	12.6–54.3	(0.44–1.67)		11.0	9.4–39.4	(0.51–1.72)	
MSI				0.5998				0.9499
yes	13.1	13.1–13.1	2.0		12.4	12.4–12.4	0.93	
no	13.9	12.3–54.3	(0.14–26.9)		11.0	9.4–39.4	(0.13–6.70)	
ECOG PS			0.44 (0.20–0.98)	0.0439			0.67 (0.38–1.17)	0.0251
0	28.4	8.4–32.7			13.3	8.8–34.9		
1	13.3	8.8–20.7			10.0	7.8–39.4		
2	10.2	9.2–39.4			8.4	6.5–32.7		
Metastatic sites			0.88 (0.35–2.19)	0.8514			0.89 (0.38–2.06)	0.9690
Liver only	17.6	7.6–29.8			14.9	6.5–20.5		
Liver + other	13.9	12.5–51.6			11.0	10.0–34.9		
Others	12.8	11.4–54.3			9.6	8.3–39.4		
Prior Lines of treatment				0.6937				0.6372
2 lines	13.7	12.5–54.3	0.85		11.0	9.4–39.4	0.84	
3 lines	13.3	8.5–39.5	(0.39–1.85)		8.4	6.5–28.4	(0.42–1.68)	

## Data Availability

The data to support the results reported in this study are available from the corresponding author upon reasonable request.
